# Cotranscription and intergenic splicing of the *PPARG *and *TSEN2 *genes in cattle

**DOI:** 10.1186/1471-2164-7-71

**Published:** 2006-04-04

**Authors:** Matthieu Roux, Hubert Levéziel, Valérie Amarger

**Affiliations:** 1 Unité de Génétique Moléculaire Animale, UMR1061 INRA/Université de Limoges, Faculté des Sciences et Techniques, 123 av Albert Thomas, 87060 Limoges Cedex, France; 2 UMR 1280 Physiologie des Adaptations Nutritionnelles, Centre INRA de Nantes, BP71627, 44316 Nantes cedex 3, France

## Abstract

**Background:**

Intergenic splicing resulting in the combination of mRNAs sequences from distinct genes is a newly identified mechanism likely to contribute to protein diversity. Few cases have been described, most of them involving neighboring genes and thus suggesting a cotranscription event presumably due to transcriptional termination bypass.

**Results:**

We identified bovine chimeric transcripts resulting from cotranscription and intergenic splicing of two neighboring genes, *PPARG *and *TSEN2*. These two genes encode the Peroxisome Proliferator Activated Receptors γ1 and γ2 and the tRNA Splicing Endonuclease 2 homolog and are situated in the same orientation about 50 kb apart on bovine chromosome 22q24. Their relative position is conserved in human and mouse. We identified two types of chimeric transcripts containing all but the last exon of the *PPARG *gene followed by all but the first exon of the *TSEN2 *gene. The two chimers differ by the presence/absence of an intermediate exon resulting from transcription of a LINE L2 sequence situated between the two genes. Both transcripts use canonical splice sites for all exons coming from both genes, as well as for the LINE L2 sequence. One of these transcripts harbors a premature STOP codon and the other encodes a putative chimeric protein combining most of the PPARγ protein and the entire TSEN2 protein, but we could not establish the existence of this protein.

**Conclusion:**

By showing that both individual and chimeric transcripts are transcribed from *PPARG *and *TSEN2*, we demonstrated regulation of transcription termination. Further, the existence and functionality of a chimeric protein harboring active motifs that are *a priori *unrelated is hypothesized.

## Background

It is now well known that diversity at the protein level can result from different positions of transcriptional start and termination of a single gene, alternative splicing and post-translational modifications. However, there is now evidence that an additional level of diversity may also exist by the production of mRNAs that combine sequence information from different genes, also known as intergenic mRNAs. Various mechanisms appear to be involved in this process and can be classified in two categories (1) trans-splicing events between pre-mRNAs of distinct genes and (2) long transcription events across neighboring genes that normally act as independent transcription units. The latter case has been described for a number of genes including *HAI-2 *and *H2RSP *[1], the *CYP2C *locus [2], *GALT *and *IL-11Rα *[3], *P2Y11 *and *SSF1 *[4], *UEV1 *and *Kua *[5], 4E-BP3 and MASK [6]. In all these cases, chimeric mRNAs that are produced might encode proteins harboring functional domains that are usually not encountered on a single protein. However, although the chimeric transcripts appear to be present at a comparable level as single gene transcripts, the *in vivo *existence of chimeric proteins has not been confirmed so far, suggesting that the translation of chimeric transcripts might be subjected to a narrow regulation.

We describe here the existence of bovine chimeric transcripts involving the Peroxisome Proliferator Activated Receptor Gamma (*PPARG*) gene and the neighboring gene, recently identified in human as the *TSEN2 *gene (tRNA Splicing Endonuclease 2 homolog) [7]. These two genes are situated 50 kb from each other on human chromosome 3p25 and their respective position is conserved on bovine chromosome 22q24 (this paper, [8]). The *PPARG *gene has been intensively studied because it encodes two nuclear receptors (PPARγ1 and PPARγ2) implicated in the regulation of a variety of cellular processes, such as cell cycle control, carcinogenesis, inflammation, atherosclerosis and mostly adipogenesis [9-11]. The reason why we decided to study this gene in cattle is because of its presumed implication in the amount of adipose tissue associated to the meat. The *TSEN2 *gene encodes a protein involved in the formation of a human endonuclease complex whose function is the removal of introns from pre-tRNAs,. The complex is a heterotretramer formed by four distinct proteins that are well characterized in yeast [12] but only very recently identified in human [7]. We identified two types of chimeric transcripts containing all but the last exon of the *PPARG *gene followed by all the coding exons of the *TSEN2 *gene (exons 2 to 12). One chimeric transcript harbors a continuous open reading frame resulting from a perfect connection of the two coding regions, but lacks the region encoded by the last exon of *PPARG*. The other chimeric transcript has an additional exon between the two regions, resulting from the transcription of a repetitive sequence situated in the 5' region of the *TSEN2 *gene and introducing a stop codon in the coding sequence.

We show here that, in cattle, both genes can be expressed as independent transcriptional units or as a hybrid *PPARG-TSEN2 *transcript devoid of the last exon of *PPARG *and the first exon of *TSEN2*. This chimeric transcript harbors an open reading frame encoding a putative chimeric protein combining domains from the two distinct proteins but we could not show any evidence of the existence of this protein. Chimeric transcripts between these two genes cannot be detected in human or mouse.

## Results

### The *PPARG *and *TSEN2 *genes form chimeric transcripts in cattle

**Figure 1:**
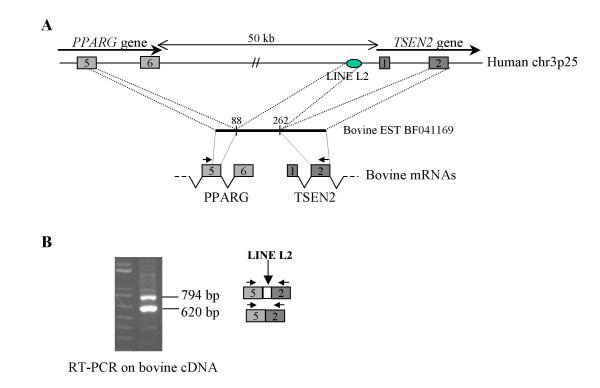
Identification of chimeric transcripts between the bovine *PPARG *and *TSEN2 *genes. A-Correspondance between the bovine EST BF041169 and human chr3p25 genomic sequence and bovine mRNAs. B- RT-PCR experiment performed on bovine cDNA using primers designed on *PPARG *exon 5 and *TSEN2 *exon 2: structure of the two amplified fragments.

When searching in the bovine EST database for ESTs corresponding to the *PPARG *gene, we found one EST sequence [Genbank:BF041169] containing the end of *PPARG *exon 5 followed by a sequence completely unrelated to the *PPARG *gene. A BLAST search with this sequence revealed a high level of identity with two regions on the human genome : (1) the 174 bp situated downstream the *PPARG *exon 5 on the EST show 76% identity with a short sequence situated 50 kb downstream the *PPARG *gene on human chromosome 3 and (2) the next 215 bp show 72% identity with the second exon of the *TSEN2 *gene, situated 6258 bp further down on the same chromosomal region (Figure 1A). The exon junctions are perfectly conserved for *PPARG *exon 5 and *TSEN2 *exon 2 and the 174 bp region between the two exons on the EST sequence appears to correspond on the human genome to a LINE L2 interspersed repeat.

The *TSEN2 *gene [Genbank:NM_025265] was recently identified in human [7]. It includes 12 exons spanning approximately 50 kb on human chromosome 3p25 and encodes a 465 amino acid protein. The start codon is situated in exon 2. This gene was not characterized in cattle. We performed a BLAST search against the bovine EST database using the human mRNA sequence as target and identified several bovine ESTs corresponding to parts of the *TSEN2 *gene [Genbank:CK963264, Genbank:AV662447, Genbank:AW358633, Genbank:AW358637]. Primers were designed from these EST sequences and RT-PCR experiments were performed in order to confirm the existence of this transcript and get the full cDNA sequence. A 1450 bp cDNA sequence [Genbank:DQ256761] was amplified using total RNA from various bovine tissues including liver, brain, skeletal muscles, spleen and heart, suggesting the ubiquitous expression of the gene. The bovine *TSEN2 *cDNA shows 77% sequence identity with the human cDNA from exon 2 to exon 12, which correspond to the coding sequence. It encodes a 459 amino acid protein displaying 72% sequence identity with the human protein. The untranslated exon 1 is not conserved between human and cattle (see below).

**Figure 2:**
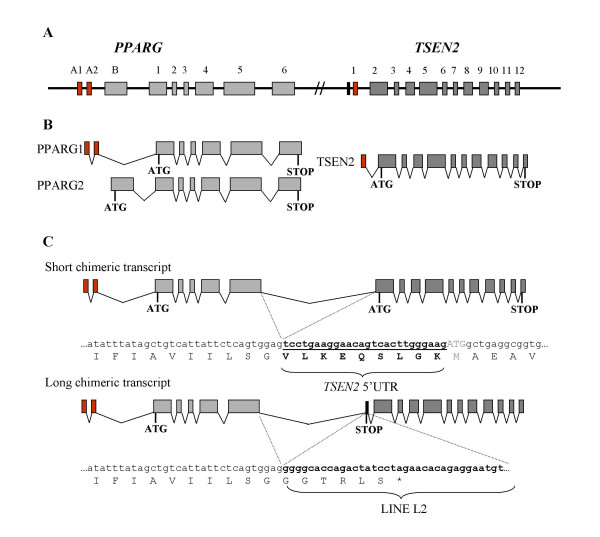
Structure of the bovine *PPARG *and *TSEN2 *genes and transcripts. A- Genomic structure of the two genes. B- Structure of the normal transcripts from both genes. C- Structure of the short and long chimeric transcripts and partial coding sequence at the junction between the two genes (untranslated exons are in red, translation start and stop are indicated).

In order to confirm the observation of the chimeric EST, we performed RT-PCR experiments on bovine cDNA using primers designed on *PPARG *exon 5 and *TSEN2 *exon 2. RT-PCR was first performed on cDNA from bovine subcutaneous adipose tissue (Figure 1B). Two different PCR products were obtained and sequenced. Both fragments contained *PPARG *exon 5 followed by *TSEN2 *exon 2 and differed in the presence/absence of the 174 bp observed on the EST BF041169 and identified as a LINE L2 repeat. We then used primers from different regions of both genes in order to establish if these chimeric transcripts contain the whole coding sequence of *PPARG *and *TSEN2 *genes. We amplified two types of chimeric transcripts both containing *PPARG *exon 1 to 5 and *TSEN2 *exon 2 to 12, and differing by the presence or absence of the LINE L2 repeat between *PPARG *exon 5 and *TSEN2 *exon 2 (referred to as long and short chimeric transcripts, Genbank:DQ256760 and Genbank:DQ256759) (Figure 2). Both types of transcripts use canonical splice sites for all exons coming from both genes.

### The chimeric transcripts might be specifically expressed from the *PPARG1 *promoter

Two different transcripts differing in their 5' end are expressed from the *PPARG *gene: *PPARG1 *(exons A1, A2, and 1 to 6) and *PPARG2 *(exon B and 1 to 6). These two transcripts result from the existence of two promoters [8] (Figure 2A). We used forward primers specific of the *PPARG1 *or *PPARG2 *transcripts together with a reverse primer in the exon 2 of the *TSEN2 *gene to test if the chimeric transcripts are expressed from both promoters. We did amplify transcripts spanning from *PPARG *exon A1 to *TSEN2 *exon 2 but we failed to amplify any RT-PCR product spanning from *PPARG *exon B to *TSEN2 *exon 2. The experiment was repeated several times with different primer sets, using primers that allowed the amplification of the individual gene transcripts, and the results were always the same.

### Chimeric transcripts encode a putative truncated PPARG protein and a putative chimeric PPARG-TSEN2 protein

Both types of chimeric transcripts have an open reading frame using the start codon from *PPARG *exon 1 and lack the STOP codon of the *PPARG *gene situated in exon 6, the only exon missing in the chimeric transcripts. In the short chimeric transcript, the open reading frame is not interrupted between *PPARG *exon 5 and *TSEN2 *exon 2 and continues to the STOP codon in *TSEN2 *exon 12 (Figure 2C). The first 26 nucleotides of *TSEN2 *exon 2 are not included in the normal coding region of the gene, but they are perfectly inserted in frame with the coding sequence of the *PPARG *gene. *PPARG *exon 5 is normally interrupted by a phase I intron (intron that interrupts a codon between the first and second nucleotide) but the connection with *TSEN2 *exon 2 restores the codon and creates a short region encoding 9 amino acids in frame with the coding region of the *TSEN2 *gene. Consequently, the short chimeric transcript encodes a putative chimeric protein of 861 amino acids containing most of the PPARγ1 protein and all the Sen2 protein, plus nine additional amino-acids separating them.

In the long chimeric transcript, the presence of the LINE L2 repeat between *PPARG *exon 5 and *TSEN2 *exon 2 creates a premature termination codon (Figure 2C), leading to a putative truncated PPARγ protein lacking the last 82 amino acids that are encoded by exon 6, but harboring instead 6 amino acids resulting from the translation of the first 18 nucleotides of the LINE L2. However, because of the presence of a premature termination codon, this transcript may be subjected to degradation by RNA surveillance mechanisms such as mRNA decay (NMD) before being translated.

### The organisation of the *PPARG-TSEN2 *genomic region is conserved between human chromosome 3p25 and bovine chromosome 22q24

We then decided to characterize and partially sequence the corresponding bovine genomic region in order (1) to establish if the relative position of the two genes is the same in cattle and human, (2) to determine if the LINE L2 repeat that is inserted in the long chimeric transcript is conserved and why that this short sequence is occasionally transcribed and (3) to localize the 5'untranslated exon 1 of the bovine *TSEN2 *gene. We used a combination of approaches to obtain the genomic sequence of this region. We screened a bovine BAC library with four sets of primers amplifying *PPARG *exon 5, *PPARG *exon 6, *TSEN2 *exon 2 and *TSEN2 *exon 3, respectively. Several BAC clones were identified among which one clone (660H1) was positive for the four primer sets and one clone (660A10) positive with the three last primer pairs. The fact that the 3'end of the *PPARG *gene and the 5'end of the *TSEN2 *gene are present in the same BAC clones confirm that they are close to each other in the bovine genome as in the human genome. We subcloned and sequence several regions from the BAC clones and supplemented them using trace sequences from the bovine genome sequencing project (obtained at [13]). We obtained a 5847 bp sequence containing the LINE L2 sequence present in the chimeric transcripts, exon 1 and part of intron 1 of the *TSEN2 *gene [Genbank:DQ256762], and a 2185 bp sequence containing the end of intron 1, exon 2 and beginning of intron 2 of the *TSEN2 *gene [Genbank:DQ256763].

**Figure 3:**
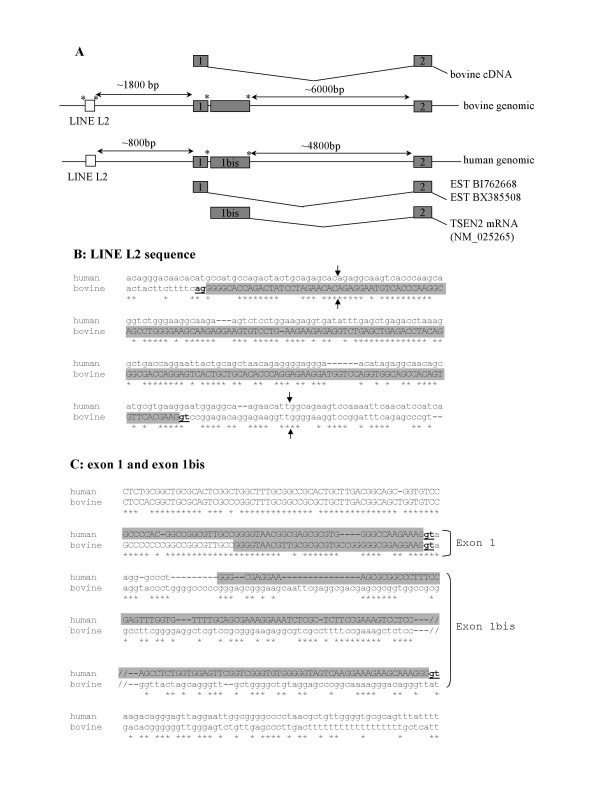
Comparison of the structure and sequence of the *TSEN2 *gene 5'end between human and cattle. A- Genomic structure of the region with the alternative untranslated exons 1 and 1bis and corresponding cDNAs. Stars indicate the presence of donor/acceptor splice sites. B- Sequence alignment of the LINE L2 repeat sequence. Arrows indicate the beginning and end of the LINE L2 as detected by Repeat Masker in both species. C- 5'untranslated exons 1 and 1bis (partial sequence) of the *TSEN2 *gene. Regions that are transcribed are outlined in grey, splice sites are in bold/underlined.

We aligned the human and bovine sequences for the 5' region of the *TSEN2 *gene. The LINE L2 repeat is conserved in sequence and position between human and cattle but there are canonical splice sites at both ends in cattle only, which might explain the fact that this sequence is present in bovine transcripts (Figure 3A–B). The bovine untranslated exon 1 present in the EST CK963264 and in our cDNA sequence is situated 1.8 kb downstream of the LINE L2 repeat. In human, there are two different exons 1 (here referred to as exon 1 and exon 1bis). Exon 1 is encountered in two human ESTs and is similar to the bovine exon 1. Exon 1bis is present in the reference mRNA sequence for the human *TSEN2 *gene [Genbank:NM_025265] and is situated 11 bp downstream of exon 1. The bovine sequence corresponding to the human exon 1bis is conserved and the canonical splice site at the 3'end (GT) is present in cattle as well (Figure 3C). However, we failed to obtain any RT-PCR product containing this exon 1bis, whereas we did amplify RT-PCR products harboring exon 1. The difference in size of the region between the conserved LINE L2 sequence and exon 1 (1.8 kb in bovine, 0.8 kb in human) is due to the presence in cattle of species-specific interspersed repeats, one LINE Bov B and two SINEs, spanning approximately 1 kb.

We compared the bovine sequence to the corresponding region in mouse (data not shown). The mouse cDNA sequence [Genbank:NM _199033] contains an untranslated exon 1 that is conserved with human and bovine exon 1. There is no evidence in the genomic sequence of a sequence that might be similar to exon 1bis. The LINE L2 repeat is not present in mouse either.

### The *PPARG *and *TSEN2 *genes are expressed in cattle as independent transcriptional units or as a hybrid *PPARG-TSEN2 *transcript

**Figure 4:**
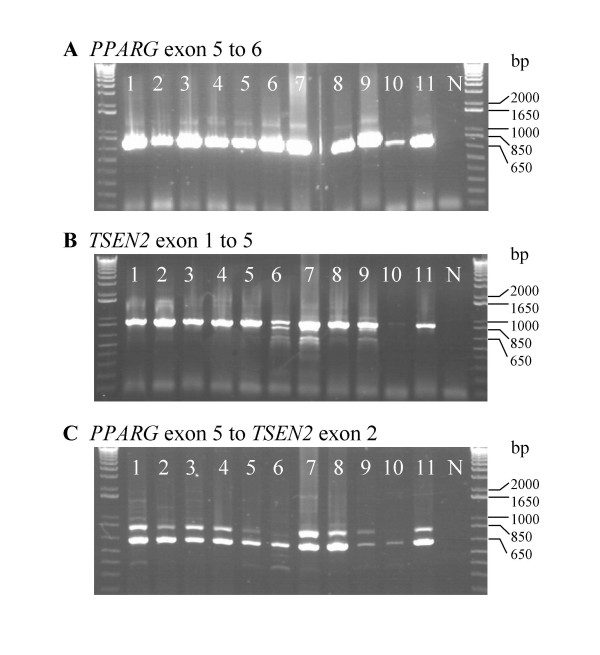
RT-PCR amplification of the *PPARG *and *TSEN2 *independent (A, B) and fusiontranscripts (C) in bovine tissues (1 to 5 skeletal muscles, 6 subcutaneous fat, 7 spleen, 8 testis,9 liver, 10 brain, 11 heart, N negative control).

We used cDNA from a set of bovine tissues to test the expression of the different transcripts. RT-PCR experiments were performed using three sets of primers specifically amplifying regions present in *PPARG *transcripts only (exon 5 to exon 6), *TSEN2 *transcripts only (exon 1 to exon 5) and chimeric transcripts only (*PPARG *exon 5 to *TSEN2 *exon 2). The expected fragments were amplified in all tissues tested (Figure 4), suggesting the ubiquitous expression of both genes as well as of the chimeric transcripts. Both long and short chimeric transcripts were present in all tissues tested but with variable proportion, the band corresponding to the long transcript being very faint in subcutaneous adipose tissue and brain. However, the same experiment was conducted on the same set of tissues from a different individual and these differences did not appear, suggesting that they may be due to individual variations and/or the quality of the RNA.

### The chimeric transcripts are expressed at a lower level compare to the *PPARG *transcripts

**Figure 5:**
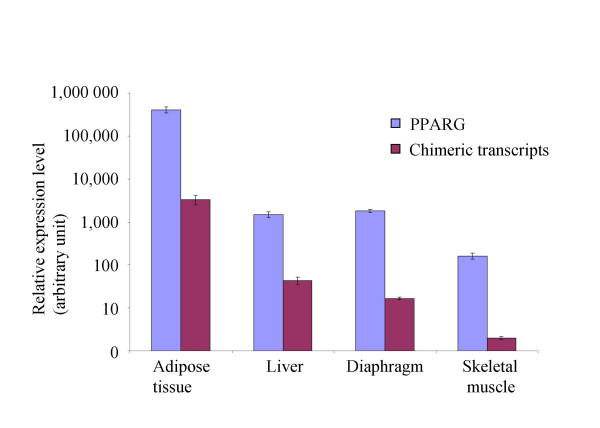
Relative expression levels of the normal *PPARG *transcripts and the chimeric transcripts assessed by semi-quantitative RT-PCR in four different tissues from one individual. Error bars stand for the variation between the values obtained from triplicates for each experiment.

We performed semi-quantitative RT-PCR experiments in order to compare the level of expression of the chimeric transcripts and the *PPARG *transcripts. Four different tissues from the same animal were tested and the relative expression of the different transcripts were normalized using the *GAPDH *gene as control. The relative expression levels of the *PPARG *transcripts (including both *PPARG1 *and *PPARG2*) and the chimeric (short and long) transcripts are presented on Figure 5. Because of the high ratio between the *PPARG *and chimeric transcripts, we used a logarithmic scale in order to be able to display both values on the same figure. This ratio varies from 124 in adipose tissue to 34 in liver, with intermediate values in muscle and diaphragm. The amount of both *PPARG *and chimeric transcripts is significantly different from one tissue to another and the ratio is not correlated to the relative level of expression. For instance, *PPARG *is expressed at a similar level in liver and diaphragm while the ratio *PPARG*/chimeric transcripts varies from 34 to 108 in these two tissues, respectively. This is most probably due to the variation in the activity of the different *PPARG *promoters from one tissue to another.

### The putative chimeric proteins are not detected on bovine tissues using immunodetection with an anti-PPARγ antibody

**Figure 6:**
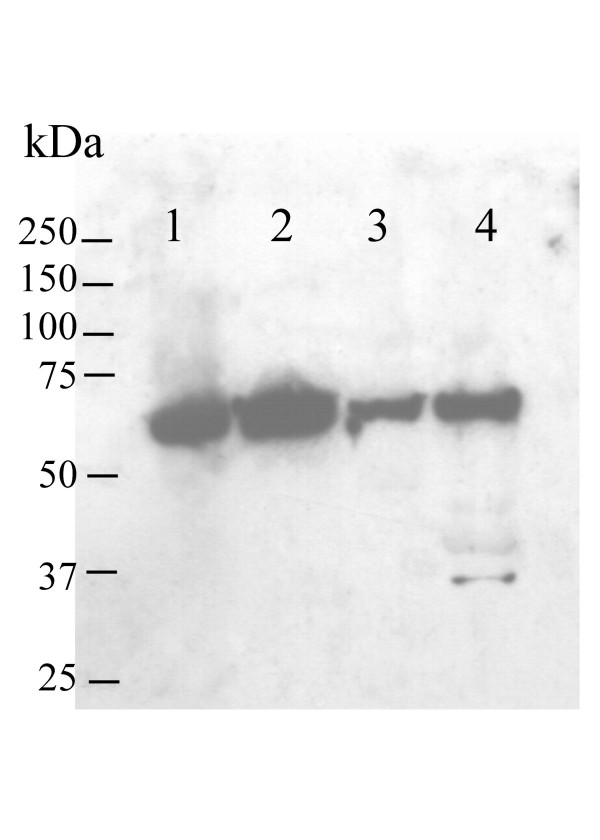
Western blot hybridization using an anti-PPARγ antibody on total protein extracts from 1- human subcutaneous adipose tissue, 2- bovine subcutaneous adipose tissue, 3- bovine brain and 4- bovine skeletal muscle.

We performed Western blot experiments using a commercial anti-PPARγ antibody. This antibody is directed against a peptide corresponding to the amino-acid residues 284–298 of mouse PPARγ2 which corresponds to a region present in both PPARγ1 and PPARγ2. This peptide is fully conserved in the human PPARγ sequence and differs only from one amino-acid with the bovine PPARγ sequence. It corresponds to a region encoded by the beginning of *PPARG *exon 5, which means that this region is present in the putative chimeric protein encoded by the short chimeric transcript. Western blots experiments were performed on proteins isolated from human and bovine subcutaneous adipose tissue, bovine brain and skeletal muscle (Figure 6). A strong signal was obtained for all tissue at the expected size for PPARγ1 and γ2 (55 to 57 kDa) but there was no evidence of a larger protein that could correspond to the chimeric protein which expected size was estimated to be around 95 kDa.

### Chimeric transcripts are not detected in human or mouse

There is no evidence in the databases of any human and mouse chimeric cDNA or EST implying the *PPARG *and *TSEN2 *genes. We performed RT-PCR experiments on cDNA isolated from human brain, adipocytes, mammary gland and skeletal muscle and a set of various mouse tissues (liver, spleen, brain, heart, skeletal muscle and subcutaneous adipose tissue) using primers designed on *PPARG *(exon 5 to 6) and *TSEN2 *(exon 1 to 2). Transcripts specific of both genes were amplified in all tissues tested and confirmed by sequencing (data not shown). However, even though we used several primer sets, we did not amplify any chimeric transcript.

In the three species, *PPARG *exon 5 ends after the first nucleotide of a codon and the junction with *TSEN2 *exon 2 would reconstitute this codon resulting in an in-frame connection between the *PPARG *and *TSEN2 *reading frames, the latter starting 26, 17 and 20 nucleotides downstream in cattle, human and mouse, respectively. However, whereas this short sequence between the two reading frames can be translated in cattle, it contains a STOP codon in human and mouse. Therefore, potential human or mouse chimeric transcripts with the same structure as bovine chimeric transcripts would anyway not encode any chimeric protein.

## Discussion

We describe here the existence of bovine chimeric transcripts resulting from intergenic splicing of two neighboring genes, *PPARG*, encoding the Peroxisome Proliferator Activated Receptors γ1 and γ2 and *TSEN2*, encoding the tRNA splicing endonuclease 2 homolog. These two genes are situated about 50 kb apart in the same 5' 3' orientation on human chromosome 3p25 and bovine chromosome 22q24. We identified two types of chimeric transcripts in cattle, differing by the presence/absence of an intermediate exon corresponding to a LINE L2 interspersed sequence situated in the 5' region of the *TSEN2 *gene. There is no evidence of such a mechanism affecting these two genes in human or mouse. Both types of chimeric transcripts contain all but the last exon of the *PPARG *gene and all but the first exon of the *TSEN2 *gene, which are normally the sites of termination and initiation of transcription, respectively. This situation is usually encountered in intergenic mRNAs resulting from a bypass of transcriptional termination. However, the reasons why this occurs are still unknown. Although this kind of event seems to be very rare, several examples have been reported, most of them affecting neighboring genes that are in the same orientation [1,3-6,14,15]. For the several cases described so far, the physical distance between the genes involved does not seem to have any influence. The genes *P2RY11 *and *PPAN *are separated by only 400 bp [4], whereas the *DISC1 *and *TRAX *genes are separated by 35 kb [14] and chimeric transcripts have been observed for genes of the *CYP2C *locus that are 26 to 85 kb apart [2].

It is important to be cautious with the interpretation of "non-conventional" RT-PCR products such as chimeric transcripts since their existence can sometimes be attributed to experimental artifacts due to template-switching activities of the reverse transcriptase (RT) or *Taq *polymerase [16]. But the structure of the chimeric molecules appears to be related to the event they derive from, as shown for the HLA class II genes [17]. Indeed, several lines of evidence suggest that the molecules we observe here are not *in vitro *artifacts. All transcripts are spliced using canonical splice sites whereas template switching activity is independent of splice sites. There is no sequence similarity that could contribute to a homology-dependent template switching. The fact that the two genes involved are adjacent to each-other and in the same orientation on the genome suggests the existence of a co-transcription unit due to a transcriptional termination bypass. Moreover, in addition to the transcripts we observed, a bovine chimeric EST is present in the EST database.

The reason why this mechanism occurs between the *PPARG *and *TSEN2 *genes in cattle but not in human and mouse is not clear. Furthermore, we observed that chimeric mRNA molecules are transcribed from the PPARγ1 promoter only. Both genes are conserved in sequence, structure, position and orientation in the three species. Sequence comparison of the PPARγ2 promoter region in human, mouse and cattle shows a high level of identity (75 to 80 % on average). The full PPARγ1 promoter genomic sequence was not available for cattle but it does not harbor any significant sequence similarity between human and mouse and the A1 exon is not conserved in sequence across the three species. This suggests that this promoter region might have species-specific functions among which is splicing regulation. Indeed, there is now evidence that the promoter structure for polymerase II plays such a role by coupling the transcription and splicing machineries (review in [18]).

The gene fusion event may also be the result of a weak termination signal for *PPARG *gene transcription. However, we demonstrate that normal *PPARG *and *TSEN2 *transcripts are also expressed, meaning that normal transcription termination and co-transcription of the two genes co-exist. Semi-quantitative RT-PCR showed that the amount of chimeric transcripts was much lower (up to 124 times lower in adipose tissue) than the amount of *PPARG *transcripts, suggesting that this phenomenon is either strictly regulated, or results from a weak leakage in the cleavage/polyadenylation of the *PPARG *transcripts. A sequence comparison of the 3'end of the *PPARG *gene in human, mouse and cattle revealed a high sequence identity between the three species. The polyadenylation site in the three species seems to be situated between a highly conserved AT-rich sequence and a reasonably conserved GT-rich sequence. These two structures constitute the most commonly found features for post-transcriptional processing. However, there is no obvious difference between the bovine and the mouse or human sequences that could explain a malfunction in cattle only. Sequence analysis shows that the reading frames of both genes cannot be joined in human and mouse in the same way they are in the bovine short chimeric transcript. This might explain why chimeric transcripts are not found in these two species and would suggest that their presence in cattle has functional significance.

We could not establish the existence of the chimeric protein in vivo. This may be due to the fact that the expression might be very low and thus be undetectable by immunodetection, as suggested by the relative quantification of the different transcripts. It is also possible that the translation of the chimeric transcripts is subjected to a strict regulation, *i.e *they are not translated at all. Indeed, although fusion transcripts for a number of genes have been reported, the existence of a fusion protein was reported only in the case of MASK-4E-BP3 [6]. The functional significance of a chimeric protein resulting from the translation of a fusion transcript between the *PPARG *and *TSEN2 *genes is difficult to assess since these two genes have specific functions that do not have *a priori *anything in common. The fusion protein would contain the first 424 amino acids from PPARγ1, the complete Sen2 protein and an intermediate region of 9 amino-acids resulting from the translation of the normally-untranslated part of *TSEN2 *exon 2. Such protein would include the DNA-binding domain and part of the ligand-binding domain of PPARγ1 (missing only the 82 amino-acids encoded by exon 6) and the tRNA intron endonuclease catalytic C terminal domain of Sen2. The Sen2 protein binds to Sen34, Sen15 and Sen54, forming the tRNA endonuclease complex and was also shown to form complexes with Clp1, which is a member of the human pre-mRNA 3'end processing complex, involved in the cleavage/polyadenylation of pre-mRNAs [7]. It is puzzling to see that *TSEN2 *appears to be involved in a phenomenon presumably due to a malfunction in the mechanisms of pre-mRNA maturation, while it is precisely one of the functions of the protein encoded by this gene.

The existence of fusion transcripts between unrelated genes is often referred to as a pathological phenomenon resulting, for example, from chromosomal translocations responsible for deregulation of gene expression and oncogene activation [19]. But is is tempting to speculate that non-conventional transcripts may encode new proteins with new functions. Hybrids between heterologous mRNAs, generated by mRNA trans-splicing or transcription of long mRNA across neighbouring loci may provide the cell with the potential for evolution and adaptation by acquiring new functions. With the increasing availability of sequence data for genomes and transcripts, it is likely that new cases of chimeric mRNA molecules will be found. An *in-silico *based approach on the UNIGENE database revealed that ~1% of the transcripts are chimeric [20]. Non-linear splice forms can also be generated by rearrangement or repetition in exon order (RREO), leading to either shorter reading frames or repetition of a given domain [21]. These computer-based studies probably give an over-estimate of the number of such non-conventional mRNA molecules because of the presence of artefactual sequences, and their sensitivity needs to be improved. However, these observations clearly suggest a biological significance of this phenomenon.

## Conclusion

Chimeric mRNA molecules resulting from cotranscription and trans-splicing of the neighboring genes *PPARG *and *TSEN2 *were indentified in cattle. The existence of these transcripts appears to be restricted to the bovine species and is probably the consequence of a modification in the transcriptional termination regulation. The functional significance of this phenomenon, if any, remains to be determined. However, this might be an example of a way for a species to increase complexity and diversity at the protein level.

## Methods

### RNA extraction and cDNA amplification and sequencing

Total RNA was isolated from bovine (about 400 to 500 mg) and mouse (C57BL/6J) tissue samples, frozen in liquid nitrogen immediately after death, using RNeasy maxi kit (Qiagen). 1 μg of RNA was reversed transcribed with oligo-dT primer and the Superscript II RT (Invitrogen). Several primer pairs were designed on EST sequences and used to amplify from the cDNA. PCR reactions were carried out in total volumes of 20 μl containing 1/10^th ^of the RT reaction, 0.2 mM dNTPs, 1.5 mM MgCl_2_, 10 pmol of each primer, UptiTherm DNA polymerase and reaction buffer (Interchim). The cycling conditions included an initial incubation at 94°C for 2 min followed by 35 cycles of 30 sec at 94°C, 30 sec at 58°C and 1 min 30 at 72°C. In some cases, touchdown PCR conditions were used. In these cases, the annealing temperature was decreased of one degree per cycle for the first seven cycles and then maintained constant for the next cycles. PCR products were purified using the QIAquick PCR purification kit (Qiagen) and directly sequenced using Big Dye Terminator chemistry (Applied Biosystems) on a ABI PRISM Cycle sequencing 3100 (Applied Biosystems).

Human cDNA was obtained by reverse transcription of commercially available RNA from human adipocytes, brain, mammary gland and skeletal muscle (Clontech).

### Semi-quantitative RT-PCR

Total RNA was isolated from bovine tissues (adipocytes, liver, diaphragm and *Rectus abdominalis *muscle) using RNeasy maxi kit (Qiagen). 1 μg of RNA was reverse transcribed using the High-Capacity cDNA Archive kit (Applied Biosystems). Quantification of *PPARG *and chimeric transcripts from bovine tissues was performed using SYBR Green (Applied Biosystems) on a ABI Prism 7900HT Sequence Detection System (Applied Biosystems) in a 20 μl reactions with 2X SYBR Green Master Mix, 300 nM of each primer and 150 ng RNA. Forward and reverse primers were placed on two consecutive exons ; primers sequences are AGCCCTTTGGTGACTTTATGGA (forward primer in exon 5) and TCCTCAATGGGCTTCACGTT (reverse primer in exon 6) for *PPARG *amplification and TGAAGTTCAACGCACTGGAATT (forward primer in exon 5 of *PPARG*) and CTGTCGTGCACTCTTCTCTTCCT (reverse primer in exon 2 of *TSEN2*) for chimeric transcripts amplification. The reaction conditions involved denaturation for 10 min at 95°C, and 40 cycles of amplification with 15 sec at 95°C and 1 min at 60°C. All samples were analyzed in triplicates, and the data were normalized using the glyceraldehyde-3-phosphate dehydrogenase gene (*GAPDH*) gene as an internal control.

### BAC subcloning and sequencing

BAC clones containing the *PPARG *and *TSEN2 *genes were obtained from the INRA bovine BAC library [22]. BAC DNA was digested by several restriction enzymes (*Eco*R I, *Hind *III, *Bam*H I) and suitable fragments were detected by Southern blot using radiolabelled probes corresponding to *TSEN2 *exon 1 and 2 and to the LINE L2 intergenic sequence. The fragments were then gel purified, subcloned into pUC18 vector and sequenced.

### Western blots

Total protein extract were prepared from tissue samples stored at -80°C as described by [23]. Human subcutaneous adipose tissue was removed from normal human skin obtained from abdominal plastic surgery. Protein content was determined by the Bradford method using BSA as standard [24]. Total proteins were analyzed by 10% SDS-PAGE. Gels were electroblotted onto nitro-cellulose membranes, probed with a polyclonal anti-PPARγ1 and γ2 antibody (Calbiochem) and visualised using BM chemiluminescence Blotting Substrate (Roche).

## Authors' contributions

MR and VA performed RNA extraction, RT-PCR and sequencing. MR performed the Western blots and real-time RT-PCR and VA the BAC subcloning and sequencing. VA conducted the study and drafted the manuscript. HL is the head of the laboratory and principal investigator of a larger project on bovine meat characteristics which led to this study.
